# The Use of Postnatal Weight Gain Algorithms to Predict Severe or Type 1 Retinopathy of Prematurity: A Systematic Review and Meta-analysis

**DOI:** 10.1001/jamanetworkopen.2021.35879

**Published:** 2021-11-23

**Authors:** Sam Athikarisamy, Saumil Desai, Sanjay Patole, Shripada Rao, Karen Simmer, Geoffrey C. Lam

**Affiliations:** 1 Neonatal Directorate, Perth Children’s Hospital and King Edward Memorial Hospital for Women, Perth, Australia; 2 School of Medicine, University of Western Australia, Crawley, Australia; 3 Department of Ophthalmology, Perth Children’s Hospital, Perth, Australia; 4 Centre for Ophthalmology and Visual Science, University of Western Australia, Crawley, Australia

## Abstract

**Importance:**

The currently recommended method for screening for retinopathy of prematurity (ROP) is binocular indirect ophthalmoscopy, which requires frequent eye examinations entailing a heavy clinical workload. Weight gain–based algorithms have the potential to minimize the need for binocular indirect ophthalmoscopy and have been evaluated in different setups with variable results to predict type 1 or severe ROP.

**Objective:**

To synthesize evidence regarding the ability of postnatal weight gain–based algorithms to predict type 1 or severe ROP.

**Data Sources:**

PubMed, MEDLINE, Embase, and the Cochrane Library databases were searched to identify studies published between January 2000 and August 2021.

**Study Selection:**

Prospective and retrospective studies evaluating the ability of these algorithms to predict type 1 or severe ROP were included.

**Data Extraction and Synthesis:**

Two reviewers independently extracted data. This meta-analysis was performed according to the Cochrane guidelines and reported according to the Preferred Reporting Items for Systematic Reviews and Meta-analysis of Diagnostic Test Accuracy Studies (PRISMA-DTA) guidelines.

**Main Outcomes and Measures:**

Ability of algorithms to predict type 1 or sever ROP was measured using statistical indices (pooled sensitivity, specificity, and summary area under the receiver operating characteristic curves, as well as pooled negative likelihood ratios and positive likelihood ratios and diagnostic odds ratios).

**Results:**

A total of 61 studies (>37 000 infants) were included in the meta-analysis. The pooled estimates for sensitivity and specificity, respectively, were 0.89 (95% CI, 0.85-0.92) and 0.57 (95% CI, 0.51-0.63) for WINROP (Weight, IGF-1 [insulinlike growth factor 1], Neonatal, ROP), 1.00 (95% CI, 0.88-1.00) and 0.60 (95% CI, 0.15-0.93) for G-ROP (Postnatal Growth and ROP), 0.95 (95% CI, 0.71-0.99) and 0.52 (95% CI, 0.36-0.68) for CHOP ROP (Children’s Hospital of Philadelphia ROP), 0.99 (95% CI, 0.73-1.00) and 0.49 (95% CI, 0.03-0.74) for ROPScore, 0.98 (95% CI, 0.94-0.99) and 0.35 (95% CI, 0.22-0.51) for CO-ROP (Colorado ROP). The original PINT (Premature Infants in Need of Transfusion) ROP study reported a sensitivity of 0.98 (95% CI, 0.91-0.99) and a specificity of 0.36 (95% CI, 0.30-0.42). The pooled negative likelihood ratios were 0.19 (95% CI, 0.13-0.27) for WINROP, 0.0 (95% CI, 0.00-0.32) for G-ROP, 0.10 (95% CI, 0.02-0.53) for CHOP ROP, 0.03 (95% CI, 0.00-0.77) for ROPScore, and 0.07 (95% CI, 0.03-0.16) for CO-ROP. The pooled positive likelihood ratios were 2.1 (95% CI, 1.8-2.4) for WINROP, 2.5 (95% CI, 0.7-9.1) for G-ROP, 2.0 (95% CI, 1.5-2.6) for CHOP ROP, 1.9 (95% CI, 1.1-3.3) for ROPScore, and 1.5 (95% CI, 1.2-1.9) for CO-ROP.

**Conclusions and Relevance:**

This study suggests that weight gain–based algorithms have adequate sensitivity and negative likelihood ratios to provide reasonable certainty in ruling out type 1 ROP or severe ROP. Given the implications of missing even a single case of severe ROP, algorithms with very high sensitivity (close to 100%) and low negative likelihood ratios (close to zero) need to be chosen to safely reduce the number of unnecessary examinations in infants at lower risk of severe ROP.

## Key Points

**Question:**

Do postnatal weight gain–based algorithms have the potential to identify infants with type 1 retinopathy of prematurity (ROP) or severe ROP?

**Findings:**

This systematic review and meta-analysis that included 61 studies (>37 000 infants) found that weight gain–based algorithms have adequate sensitivity, ranging from 0.89 to 1.00, and negative likelihood ratios (<0.2). However, specificity and positive likelihood ratios were inadequate.

**Meaning:**

This study suggests that weight gain–based algorithms have adequate sensitivity and negative likelihood ratios and provide reasonable certainty that type 1 ROP or severe ROP is unlikely to develop (ie, the algorithm is useful for ruling out the disease).

## Teaser

This systematic review and meta-analysis of 61 studies including more than 30 000 infants synthesizes evidence regarding the ability of postnatal weight gain–based algorithms to predict type 1 or severe retinopathy of prematurity.

## Introduction

Retinopathy of prematurity (ROP) is a disease of pathologic neovascularization affecting preterm infants. In early postnatal life, hyperoxia leads to suppression of vascular growth factors (phase 1). Subsequently, as retinal hypoxia sets in, there is an upsurge of vascular growth factors leading to unregulated vasoproliferation (phase 2).^1^ ROP either regresses spontaneously or continues to advance and can progress to cause retinal detachment and blindness if not detected and treated early.^2^ Infants with lower gestation and lower birth weight have a higher risk of developing ROP. Currently, at-risk infants are screened using repeated eye examinations (binocular indirect ophthalmoscopy [BIO]) starting at approximately 30 to 32 weeks’ postmenstrual age and continuing until the retinal vasculature is fully mature (approximately 40 weeks’ postmenstrual age).^3^ However, only fewer than 10% of screened infants need treatment for ROP.^2^

Animal experiments have demonstrated the importance of nutrition and insulinlike growth factor 1 (IGF-1) in the retinal vascular development. Insufficient activation of endothelial growth factor by IGF-1 can alter the development of the retinal vasculature.^4^ In the clinical setting, low postnatal weight gain is considered as a surrogate marker for slower-than-expected increases in serum IGF-1 levels.^5^ Based on this hypothesis, risk prediction models such as WINROP (Weight, IGF-1, Neonatal Retinopathy of Prematurity),^6^ G-ROP (Postnatal Growth and Retinopathy of Prematurity),^7^ PINT (Premature Infants in Need of Transfusion) ROP,^8^ CHOP (Children's Hospital of Philadelphia) ROP,^9^ ROPScore,^10^ and CO-ROP (Colorado Retinopathy of Prematurity),^11^ have been evaluated to see whether they can predict the development of significant ROP. These models have the potential advantage of reducing the number of BIO examinations. However, to our knowledge, these models have not been implemented in clinical practice because of their limited generalizability.^3^

The rationale for this systematic review was to assess whether postnatal weight gain–based algorithms have the potential to predict the development of type 1 or severe ROP. This review gains further importance given the current COVID-19 pandemic, which has led to the curtailment of health services, including ROP screening programs, due to the limited availability of ophthalmologists and mobile screening teams.^12^ The aim of this systematic review was to synthesize evidence by pooling the diagnostic accuracy indices for postnatal weight gain–based algorithms for predicting type 1 or severe ROP in preterm infants.

## Methods

### Design and Registration

This meta-analysis was performed according to the Cochrane guidelines^13^ and reported according to the Preferred Reporting Items for Systematic Reviews and Meta-analysis of Diagnostic Test Accuracy Studies (PRISMA-DTA) guidelines.^14^ The study protocol was registered in PROSPERO (CRD42020172874).^15^

### Search Strategy

A systematic search of the PubMed, MEDLINE, Embase, and Cochrane Library databases was performed to identify studies published between January 2000 and August 2021. PubMed was searched using the standard terminology (eMethods in the Supplement). Similar terminology was used while searching other databases. We also searched the Cochrane Library, ClinicalTrials.gov, grey literature (on OpenGrey, Google Scholar, and MedNar). No language restrictions were applied. The reference lists of all publications were searched manually for additional studies.

### Data Extraction

Two reviewers (S.A. and S.D.) independently collected data from the included studies. Study data were further verified by one of the reviewers (S.R.) who had not collected the study data earlier. A total of 61 studies were included for the final meta-analysis. The following data were collected from each study: year(s) the study was conducted, country, gestational age at birth, birth weight, weekly weight gain, total sample size, follow-up rates, tool used to diagnose ROP (indirect ophthalmoscopy or wide-field digital retinal imaging), prospective or retrospective design, high-income or low- to middle-income country (World Bank list of economies),^16^ and diagnostic indices (true positives, false positives, true negatives, and false negatives). The process of study selection is shown in the study flow diagram (eFigure 1 in the Supplement).

### Criteria for Considering Studies for This Review

The outcomes examined were the sensitivity and specificity of postnatal weight gain–based algorithms to predict type 1 or severe ROP.^2^ Studies that used type 1 ROP or severe ROP as the target disease were included.

Both prospective and retrospective studies that met the following criteria were included: (1) retinopathy screening for preterm infants and (2) the ability of weight gain–based screening algorithms to predict type 1 or severe ROP. The following standard definitions were accepted for type 1 ROP^2^: zone I (any stage ROP with plus disease), zone I (stage 3 with or without plus disease), or zone II (stage 2 or 3 ROP with plus disease).

Severe ROP was defined as any ROP in zone I, stage 2 ROP in zone II with plus disease, or any stage 3 ROP. The studies included described the diagnostic ability of weight gain–based algorithms to predict type 1 or severe ROP in comparison with the findings by ophthalmologists either by BIO or by wide-field digital retinal imaging, which were considered the reference standards.^3^ Two reviewers (S.A. and S.D.) independently decided on the eligibility of studies for inclusion in the systematic review. Differences in opinion were resolved by discussion among all reviewers.

### Quality Assessment

The quality assessment of included studies consisted of the following 4 domains according to the revised version of the Quality Assessment of Diagnostic Accuracy Studies (QUADAS-2): patient selection, index test, reference standard, and flow and timing.^17^ In this review, index test refers to the “alarm” in any of the postnatal weight gain–based algorithms for type 1 or severe ROP. The included studies were assessed for risk of bias in each domain and for applicability concerns in the first 3 domains.

### Statistical Analysis

Meta-analysis was conducted using STATA, version 16 (StataCorp)^18^ by one of us (S.R.) in the presence of 2 other authors (S.A. and S.D.). We used the bivariate mixed-effects regression model to derive the pooled sensitivity, specificity, positive likelihood ratio (PLR), and negative likelihood ratio (NLR) and the diagnostic odds ratio (DOR) and respective 95% CIs. A summary receiver operating characteristic (ROC) curve was generated to display the results of individual studies. The following cutoffs were used for interpretation of the area under the ROC values: low (0.5-0.7), moderate (0.71-0.9), or high (>0.9) accuracy.^19^ The DOR is the ratio of the odds of positivity in the disease relative to the odds of positivity in those without the disease. The value of the DOR ranges from 0 to infinity, with higher values indicating better discriminatory test performance. A likelihood ratio of approximately 1 means the test result neither rules in nor rules out the condition. A PLR above 1 indicates increased evidence of disease; the higher from 1, the more chance of disease. An NLR below 0.1 is very strong evidence to rule out a disease.^20^

### Additional Analysis

There is uneven practice^21^ with regard to oxygen saturation targeting, blood transfusion thresholds, and higher incidence of fetal growth restriction in low- and middle-income countries; these factors could have an impact on the performance of these algorithms. Hence, sensitivity analyses were conducted separately for high-income and low- and middle-income countries when possible.

## Results

### Selected Studies

The electronic database search yielded 779 titles and abstracts and 3 additional studies by exploring additional sources. After removal of duplicates, 160 articles were screened based on the title and abstract, and 85 articles were selected. The full text was read for eligibility, of which 61 were included in the final analysis. The 61 studies (>37 000 infants) included WINROP (n = 36),^6,22,23,24,25,26,27,28,29,30,31,32,33,34,35,36,37,38,39,40,41,42,43,44,45,46,47,48,49,50,51,52,53,54,55^ G-ROP (n = 9),^7,42,56,57,58,59,60,61^ PINT ROP (n = 1),^8^ CHOP ROP (n = 6),^9,30,55,62,63,64^ ROPScore (n = 5),^10,30,55,65,66^ and CO-ROP (n = 4).^11,67,68,69^ Studies that used a different cutoff score for ROPScore were not included in the analysis.^64,70^

The general characteristics of the studies included in the systematic review are reported in eTables 1 to 6 in the Supplement. The methodological quality and applicability of the included studies were assessed according to the QUADAS-2 guidelines.^17^ All 61 studies evaluated one of the weight gain–based algorithms, and there was minimal risk of bias. However, the reference standards used in the studies were either BIO or wide-field digital retinal imaging.

### Meta-analysis

#### WINROP

**Figure 1.**
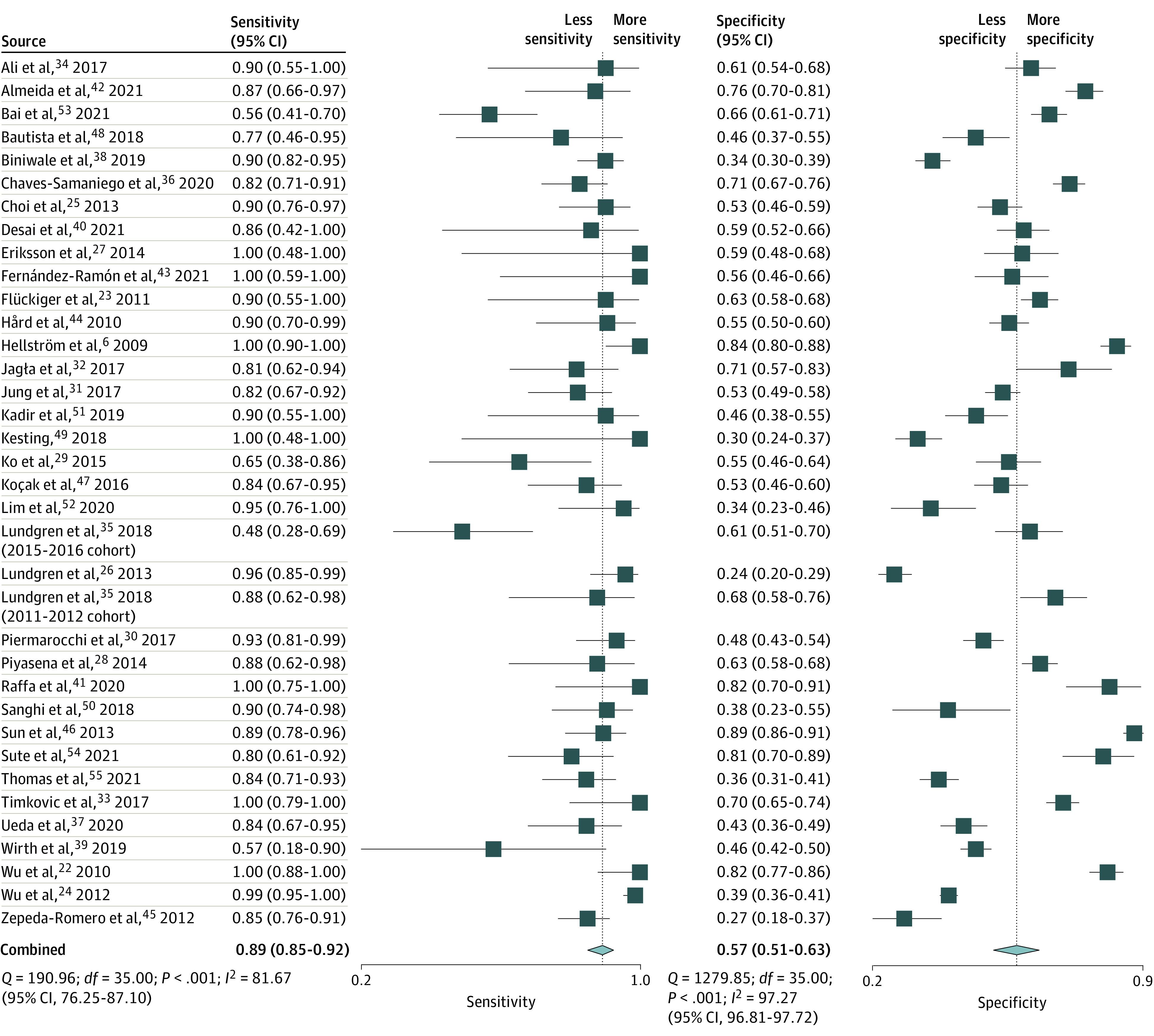
Pooled Estimate for Sensitivity and Specificity of the Weight, Insulinlike Growth Factor 1, Neonatal Retinopathy of Prematurity (WINROP) Algorithm

**Figure 2.**
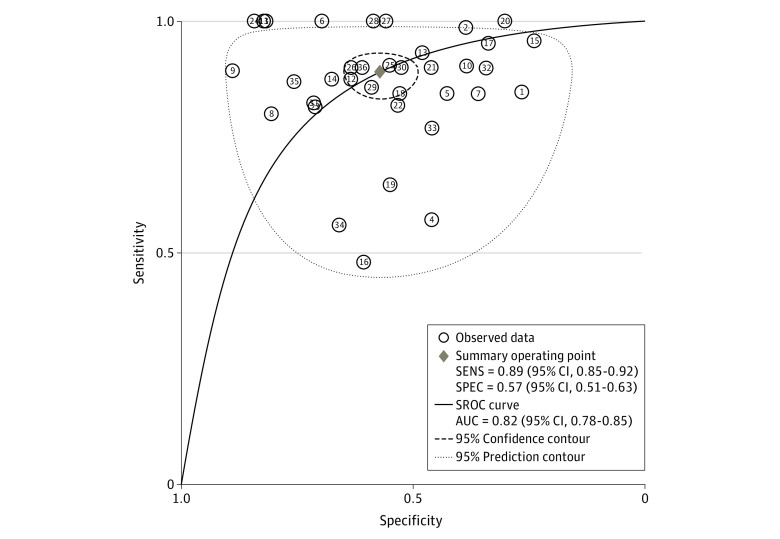
Summary Area Under the Receiver Operating Characteristic Curve of Weight, Insulinlike Growth Factor 1, Neonatal Retinopathy of Prematurity (WINROP) Algorithm AUC indicates area under the curve; SENS, sensitivity; SPEC, specificity; and SROC, summary receiver operating characteristic.

**Figure 3.**
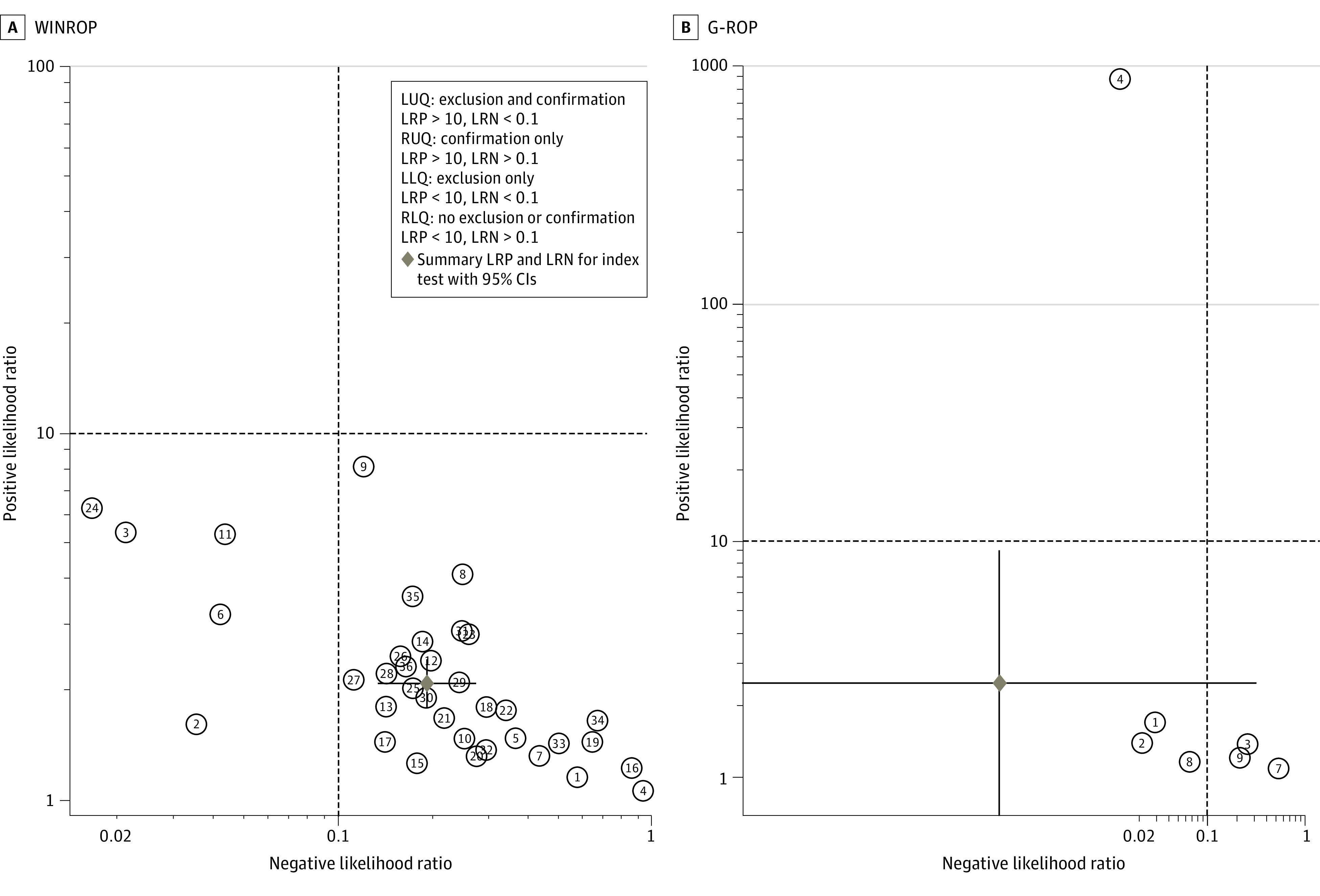
Likelihood Matrix A, Summary positive likelihood ratio (PLR) and negative likelihood ratio (NLR) for Weight, Insulinlike Growth Factor 1, Neonatal Retinopathy of Prematurity (WINROP) are shown in the right lower quadrant (RLQ). B, Summary PLR and NLR for Postnatal Growth and Retinopathy of Prematurity (G-ROP) are shown in the RLQ. LLQ indicates left lower quadrant; LRN, likelihood ratio negative; LRP, likelihood ratio positive; LUQ, left upper quadrant; and RUQ, right upper quadrant.

The pooled estimates from 36 studies (n = 11 500) for the sensitivity of the WINROP algorithm to predict type 1 or severe ROP was 0.89 (95% CI, 0.85-0.92), and the pooled estimate of the specificity was 0.57 (95% CI, 0.51-0.63) (Figure 1; Table). The summary area under the ROC curve was 0.82 (95% CI, 0.78-0.85) (Figure 2). The pooled PLR was 2.1 (95% CI, 1.8-2.4), and the pooled NLR was 0.19 (95% CI, 0.13-0.27) (eFigure 2 in the Supplement). The summary PLR and NLR for index test fell in the right lower quadrant of the likelihood matrix (Figure 3A). The pooled DOR of the WINROP algorithm was 11 (95% CI, 7-17).

**Table.** Summary of ROP Prediction Algorithms Based on Postnatal Weight Gain

Algorithm name and description	Components of algorithm	No. of studies (No. of infants)	Diagnostic indices, pooled estimates (95% CI)						Strengths	Limitations
			Sensitivity	Specificity	Positive likelihood ratio	Negative likelihood ratio	Diagnostic odds ratio	AUROC		
**WINROP** ^6^										
Cumulative deviations statistical approach: deviations between expected and actual weight accumulated week to week; expected weight data derived from control infants with no or mild ROP; alarms when cumulative deviations exceed a threshold	Gestational age, birth weight, and weekly input of observed weight	24 (8543) For high-income countries; 12 (2957) for low- to middle-income countries; 36 (11 500) for both high- and low- to middle-income countries	0.91 (0.85-0.95) For high-income countries; 0.85 (0.78-0.90) for low- to middle-income countries; 0.89 (0.85-0.92) for both high- and low- to middle-income countries	0.60 (0.53-0.66) For high-income countries; 0.51 (0.39-0.64) for low- to middle-income countries; 0.57 (0.51-0.63) for both high- and low- to middle-income countries	2.3 (1.9-2.7) For high-income countries; 1.7 (1.3-2.3) for low- to middle-income countries; 2.1 (1.8-2.4) for both high- and low- to middle-income countries	0.15 (0.08-0.26) For high-income countries; 0.29 (0.19-0.45) for low- to middle-income countries; 0.19 (0.13-0.27) for both high- and low- to middle-income countries	15 (8-30) For high-income countries; 6 (3-11) for low- to middle-income countries; 11 (7-17) for both high- and low- to middle-income countries	0.82 (0.78-0.85) For high-income countries; 0.81 (0.78-0.84) for low- to middle-income countries; 0.82 (0.78-0.85) for both high- and low- to middle-income countries	Most widely studied in both high-income and low- to middle-income countries; provides risk assessment every week through a web-based application; algorithm will often identify infants as high- or low-risk well before eye examinations are started	Complex calculation involved in the algorithm; low sensitivity reported from low- to middle-income countries
**G-ROP** ^7^										
Hybrid model; 6 components; infant qualifies for a retinal examination if any of the algorithm components is present	Gestational age <28 wk; birth weight <1051 g; weight gain over 3 growth periods (10-19 d: <120 g; 20-29 d: <180 g; and 30-39 d: <170 g); hydrocephalus	9 (14 120)	1.00 (0.88-1.00)	0.60 (0.15-0.93)	2.5 (0.7-9.1)	0.00 (0.00-0.32)	3523 (4-3 155 457)	0.99 (0.98-1.00)	Validated in a multicenter cohort with a large sample size; high sensitivity and low negative likelihood ratio	Relatively new algorithm; more studies are needed to assess generalizability
**PINT ROP** ^8^										
A simpler (logistic regression) predictive model; an alarm is triggered when the risk is >0.085 on the scale provided	Birth weight <1000 g; gestational age; daily weight gain rate (weight gain rate calculated from the current and prior week’s measurement)	1 (334)	0.98 (0.91-0.99)	0.36 (0.30-0.42)	1.55 (1.41-1.69)	0.04 (0.01-0.29)	38.75 (9.00-58.00)	NA	Simple paper-based nomogram; model evaluated risk on a weekly basis	Not widely validated
**CHOP ROP** ^9^										
A simpler (logistic regression) predictive model; similar to PINT ROP but included infants with birth weight <1501 g; an alarm is triggered when the risk is >0.014 on the scale (nomogram)	Birth weight <1501 g; gestational age; daily weight gain rate (weight gain rate calculated from the current and prior week’s measurement)	6 (2135)	0.95 (0.71-0.99)	0.52 (0.36-0.68)	2.0 (1.5- 2.6)	0.10 (0.02-0.53)	20 (4-99)	0.75 (0.71-0.79)	Simple paper-based nomogram; model evaluated risk on a weekly basis	Poor generalizability; low sensitivity reported from low- to middle-income countries
**ROPScore** ^10^										
A simpler (logistic regression) equation model (score based on cumulative risk factors); a cutoff point of 11 was established for any stage ROP and 14.5 for severe ROP	Birth weight; gestational age; weight gain at a single time point (from birth to 6 wk) as a proportion of birth weight; oxygen use on ventilator; blood transfusion	5 (1625)	0.99 (0.73-1.00)	0.49 (0.03-0.74)	1.9 (1.1-3.3)	0.03 (0.00-0.77)	69 (2-2228)	0.88 (0.84-0.90)	Takes into account other risk factors (blood transfusion, oxygen use on mechanical ventilation)	Once per child risk calculation at 6 wk of age; hence, could potentially miss infants developing aggressive posterior ROP; validated in low- to middle-income countries and not tested widely in high-income countries
**CO-ROP** ^11^										
A simple criteria; infant qualifies for retinal examination if all 3 components from the algorithms are present	Gestational age <30 wk; birth weight <1500 g; weight gain at a single time point (from birth to 4 wk) <650 g	4 (8082)	0.98 (0.94-0.99)	0.35 (0.22-0.51)	1.5 (1.2-1.9)	0.07 (0.03-0.16)	22 (9-58)	0.95 (0.93-0.97)	High sensitivity in the original cohort (100%)	Poor generalizability; low sensitivity reported in later cohorts from US and Canada

Abbreviations: AUROC, area under the receiver operating characteristic curve; CHOP ROP, Children's Hospital of Philadelphia Retinopathy of Prematurity; CO-ROP, Colorado Retinopathy of Prematurity; G-ROP, Postnatal Growth and Retinopathy of Prematurity; NA, not applicable; PINT ROP, Premature Infants in Need of Transfusion Retinopathy of Prematurity; ROP, retinopathy of prematurity; WINROP, Weight, Insulinlike Growth Factor 1, Neonatal Retinopathy of Prematurity.

#### WINROP (High-Income vs Low- to Middle-Income Countries)

The sensitivity analyses of the WINROP algorithm assessed its performance in high-income and low- to middle-income countries. For high-income countries, the pooled estimate from 24 studies (n = 8543) for the sensitivity of the WINROP algorithm was 0.91 (95% CI, 0.85-0.95), and the pooled estimate for the specificity was 0.60 (95% CI, 0.53-0.66) (Table). The summary area under the ROC curve was 0.82 (95% CI, 0.78-0.85). The PLR was 2.3 (95% CI, 1.9-2.7), and the NLR was 0.15 (95% CI, 0.08-0.26).

For low- to middle-income countries, the pooled estimate from 12 studies (n = 2957) for the sensitivity of the WINROP algorithm was 0.85 (95% CI, 0.78-0.90), and the pooled estimate for the specificity was 0.51 (95% CI, 0.39-0.64) (Table). The summary area under the ROC curve was 0.81 (95% CI, 0.78-0.84). The PLR was 1.7 (95% CI, 1.3-2.3), and the NLR was 0.29 (95% CI, 0.19-0.45).

#### G-ROP

**Figure 4.**
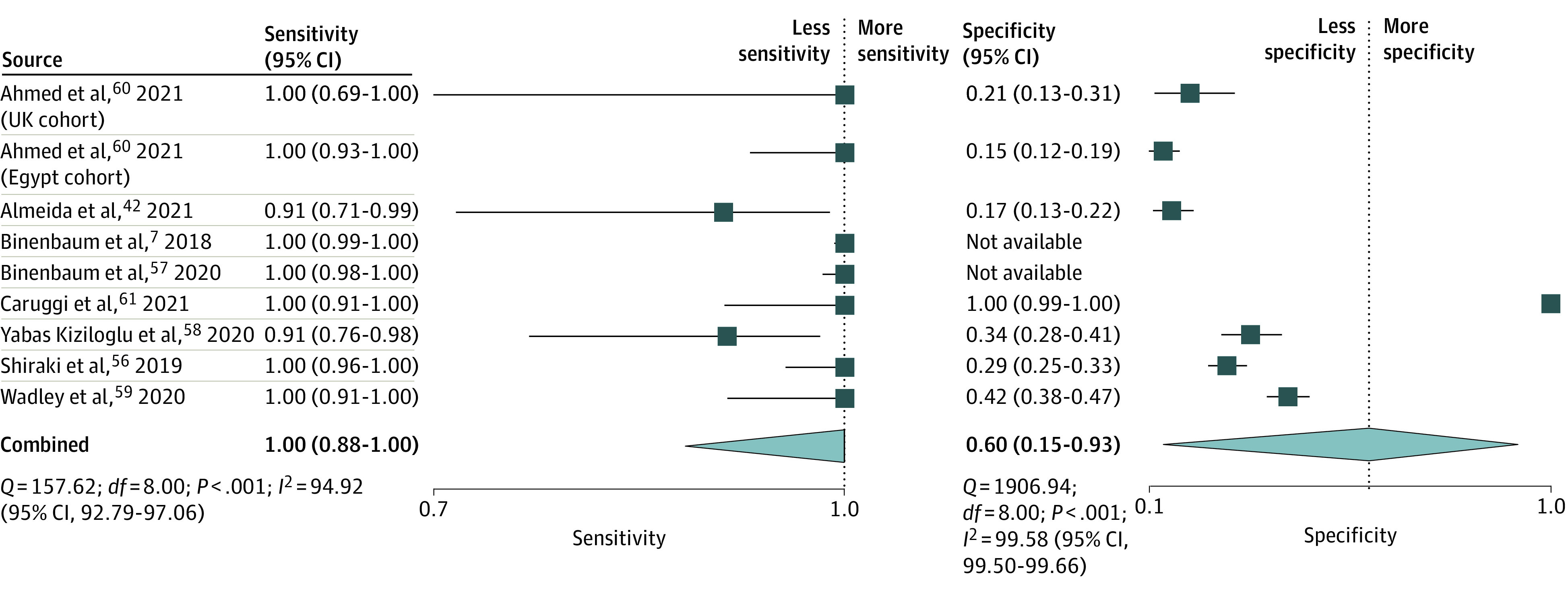
Pooled Estimate for Sensitivity and Specificity of Postnatal Growth and Retinopathy of Prematurity (G-ROP) Model

The pooled estimates from 9 studies (n = 14 120) reported a sensitivity of 1.00 (95% CI, 0.88-1.00) and a specificity (7 studies) of 0.60 (95% CI, 0.15-0.93) for G-ROP to predict type 1 or severe ROP (Figure 4; Table). The summary area under the ROC curve was 0.99 (95% CI, 0.98-1.00). The PLR was 2.5 (95% CI, 0.7-9.1), and the NLR was 0.00 (95% CI, 0.00-0.32) (eFigure 3 in the Supplement). The pooled DOR of the G-ROP algorithm to predict type 1 or severe ROP was 3523 (95% CI, 4-3 155 457). The summary PLR and NLR for index test fell in the left lower quadrant of the likelihood matrix (Figure 3B).

#### PINT ROP

The original PINT ROP study (n = 334) reported a sensitivity of 0.98 (95% CI, 0.91-0.99) and a specificity of 0.36 (95% CI, 0.30-0.42) for PINT ROP to predict type 1 or severe ROP (Table). The PLR was 1.55 (95% CI, 1.41-1.69), and the NLR was 0.04 (95% CI, 0.01-0.29). The DOR was 38.75 (95% CI, 9.00-58.00).

#### CHOP ROP

The pooled estimates from 6 studies (n = 2135) showed a sensitivity of 0.95 (95% CI, 0.71-0.99) and a specificity of 0.52 (95% CI, 0.36-0.68) for the CHOP ROP algorithm to predict type 1 or severe ROP (Table). The summary area under the ROC curve was 0.75 (95% CI 0.71-0.79). The PLR was 2.0 (95% CI, 1.5-2.6), and the NLR was 0.10 (95% CI, 0.02-0.53). The pooled DOR of the CHOP ROP algorithm was 20 (95% CI, 4-99).

#### ROPScore

The pooled estimates from 5 studies (n = 1625) showed a sensitivity of 0.99 (95% CI, 0.73-1.00) and a specificity of 0.49 (95% CI, 0.03-0.74) for ROPScore to predict type 1 or severe ROP (Table). The summary area under the ROC curve was 0.88 (95% CI, 0.84-0.90). The PLR was 1.9 (95% CI, 1.1-3.3), and the NLR was 0.03 (95% CI, 0.00-0.77). The pooled DOR of the ROPScore algorithm was 69 (95% CI, 2-2228).

#### CO-ROP

The pooled estimates from 4 studies (n = 8082) showed a sensitivity of 0.98 (95% CI, 0.94-0.99) and a specificity of 0.35 (95% CI, 0.22-0.51) for CO-ROP to predict type 1 or severe ROP (Table). The summary area under the ROC curve was 0.95 (95% CI, 0.93-0.97). The PLR was 1.5 (95% CI, 1.2-1.9), and the NLR was 0.07 (95% CI, 0.03- 0.16). The pooled DOR of the CO-ROP algorithm was 22 (95% CI, 9-58).

## Discussion

The incidence of blindness after ROP is increasing globally. Early identification and treatment of ROP are of paramount importance to prevent irreversible blindness. Current screening protocols that require frequent retinal examinations place an enormous workload on the health care system. The current COVID-19 pandemic has further strained the health care system owing to staff shortages, bed shortages, and limited availability of transport. Also, there is a risk of COVID-19 transmission to the preterm infants undergoing screening for ROP due to close contact with screening staff. The Royal Ophthalmologist College has issued a statement in this regard to overcome the challenges by rationalizing the ROP screening criteria by using evidence-based weight gain–based algorithms.^12^

Weight gain–based screening models, such as WINROP, G-ROP, PINT ROP, CHOP ROP, ROPScore, and CO-ROP,^6,7,8,9,10,11^ have been evaluated in various studies to identify infants who should be referred for retinal examination. However, the 2018 policy statement from the American Academy of Pediatrics stated that the uses of such weight gain–based algorithms alone are not justified based on the current literature.^3^ Our systematic review increases the evidence base in this area by including more than 37 000 preterm infants from 61 studies in diverse settings across the world.

The general interpretation of the results of diagnostic accuracy studies are as follows: PLRs of more than 10 or NLRs less than 0.1 generate large and often conclusive changes in the posttest probability, PLRs from more than 5 to 10 or NLRs from 0.1 to 0.2 generate moderate shifts in posttest probability, PLRs from more than 2 to 5 or NLRs from more than 0.2 to 0.5 generate small changes in posttest probability, and PLRs from more than 1 to 2 or NLRs from more than 0.5 to 1 alter posttest probability to a very small degree.^71,72^ High sensitivity corresponds to a high negative predictive value and a low NLR and is the ideal property of a “rule-out” test.^73,74^ Because all of the algorithms that we evaluated had an NLR of less than 0.2 and a high sensitivity of 0.89 to 1.00, they are useful to rule out type 1 ROP if the algorithm had “no alarm.”

Given that the sensitivity of G-ROP were better than WINROP and the sample size was larger, G-ROP may be more suitable as a rule-out test compared with WINROP. However, G-ROP is not widely evaluated outside high-income countries. High specificity corresponds to a high positive predictive value and a high PLR and is the ideal property of a “rule-in” test.^73,74^ Because all of the algorithms had a very low specificity and a very low PLR of 1.5 to 2.5, they may not be suitable for ruling in the disease.

Although the overall sensitivity (0.89) and NLR (0.19) for WINROP were reasonably adequate, they were significantly lower than the validation studies that were performed in the early 2010. The sensitivity was 1.00 in a Swedish cohort^6^ of 353 infants and a Boston cohort^22^ of 318 infants. A multicenter study in the US and Canada showed that the sensitivity of the WINROP algorithm was 0.986.^24^ The sensitivity values were lower in studies conducted in low- to middle-income countries. The publication by Lundgren et al^35^ in 2018 showed an associaton between the decrease in sensitivity and the change in oxygen target ranges that occurred in 2010. This could have been the explanation for the low sensitivity in low- to middle-income countries, where there is uneven practice with regard to oxygen targeting in preterm infants.^44,45,46,47,48,49,50,51,52,53,54,55^

The G-ROP model was originally developed from a retrospective cohort of 7483 infants from 29 North American centers that showed a sensitivity of 1.00 to identify type 1 ROP.^7^ This finding was further validated in a prospective cohort of 3981 infants in North America with a similar sensitivity of 1.00.^57^ When validated outside the North American cohort, the sensitivity was 1.00 in a small cohort of infants from Japan,^56^ the UK, and Egypt^60^; however, it decreased to approximately 0.91 in a Turkish and Portuguese cohort.^42,58^

Summary PLRs and NLRs for index tests fell in the right lower quadrant of the likelihood matrix for WINROP and the left lower quadrant for G-ROP. This finding suggests that diagnostic indices are better for G-ROP compared with WINROP. However, the strength of WINROP is that it has been tested extensively in nearly 36 studies across the world.

There are a significant number of infants in low- and middle-income countries who develop type 1 ROP and who are more mature (>28 weeks) and heavier (>1050 g).^75^ Hence, validating the G-ROP algorithm in these groups of infants will give additional information about the performance of the weight gain component of the G-ROP algorithm (weight gain over 3 growth periods: 10-19 days, 20-29 days, and 30-39 days).

The CHOP ROP model was an updated version of the PINT ROP model and had a sensitivity of 1.00 to identify type 1 ROP in the original cohort and a sensitivity of 0.985 to identify type 1 ROP in a multicenter cohort.^9,62^ Sensitivities of 0.667 and 0.549 in the CHOP ROP model were reported from 2 different Indian cohorts.^55,63^ The ROPScore had additional risk factors, such as oxygen use in mechanical ventilation and blood transfusion.^10^ In the original cohort, it had a sensitivity of 0.9583; however, when it was tested using different cutoff scores, the sensitivity was 0.50 to 1.00 in different cohorts. Proportional weight gain calculated at 6 weeks of postnatal life could potentially miss some of the infants who would develop aggressive posterior ROP within this time period.

This review summarizes the evidence for weight gain–based algorithms and their performance in predicting type 1 or severe ROP. This review has highlighted how the performance of these weight gain–based models has varied in their predictive values depending on the situation in which it was tested. Hesitancy in implementing these algorithms into clinical practice is justifiable because the focus has been on not missing a single child who may need treatment.

Postnatal weight gain–based models are not ideal for infants who show a pattern of nonphysiological weight gain (eg, hydrocephalus, sepsis, and patent ductus arteriosus). Future studies should endeavor to incorporate other risk factors, such as oxygen use and sepsis, into the algorithm to improve their diagnostic performance. Another area of research would be to validate these algorithms against a combined reference standard rather than BIO or wide-field digital retinal imaging individually.

### Strengths and Limitations

This study has some strengths. To our knowledge, this is the first systematic review including a meta-analysis to evaluate the diagnostic accuracy of postnatal weight gain–based algorithms with a sample size of more than 37 000 infants. The study sample included infants from both high-income and low- to middle-income countries, and additional analyses were performed in these subgroups. Furthermore, a standardized tool (ie, QUADAS-2) was used for the quality assessment. In addition, this systematic review and meta-analysis followed the recent PRISMA-DTA guidelines for transparent reporting.

This study also has some limitations. The weight gain–based algorithms rely strongly on accurate measurement of weight, which is very challenging in a neonatal intensive care setting and could potentially affect the performance of the algorithms. Apart from the index test, there were multiple heterogeneities present across the studies. These heterogeneities were partly explained by factors such as geographical background, workflow, and the choice of a reference standard, which in itself is known to have interobserver variability.

## Conclusions

This systematic review and meta-analysis suggests that weight gain–based algorithms have adequate sensitivity and negative likelihood ratios to provide reasonable certainty in ruling out type 1 or severe ROP. Given the implications of missing even a single case of severe ROP, algorithms with very high sensitivity (close to 100%) and low negative likelihood ratio (close to zero) need to be chosen. These algorithms have the potential to reduce the number of unnecessary examinations for infants at lower risk of severe ROP. Future studies should endeavor to incorporate additional clinical parameters (eg, oxygen use and sepsis), which could potentially improve the diagnostic indices of these algorithms.

## Notes

Supplement. **eFigure 1.** Flow Diagram for Study Selection Process **eFigure 2.** The Positive (PLR) and Negative Likelihood Ratio (NLR) for WINROP Algorithm **eFigure 3.** The Positive (PLR) and Negative Likelihood Ratio (NLR) for G-ROP Model
eMethods.
**eTable 1.** Characteristics of Included Studies of WINROP Algorithm, From High Income Countries **eTable 2.** Characteristics of Included Studies Evaluating WINROP Algorithm, From Low and Middle-Income Countries **eTable 3.** Characteristics of Included Studies Evaluating G-ROP (Postnatal Growth and ROP) **eTable 4.** Characteristics of Included Studies Evaluating CHOP ROP **eTable 5.** Characteristics of Included Studies Evaluating ROP Score Based on Cumulative Scores **eTable 6.** Characteristics of Included Studies Evaluating Colorado ROP
