# Supplementary material for: The Use of Postnatal Weight Gain Algorithms to Predict Severe or Type 1 Retinopathy of Prematurity: A Systematic Review and Meta-analysis
Source: JAMA Netw Open. 2021 Nov 23;4(11):e2135879. doi: 10.1001/jamanetworkopen.2021.35879 (PMC8611486; doi:10.1001/jamanetworkopen.2021.35879)
Supplement: Supplement. — eFigure 1. Flow Diagram for Study Selection Process eFigure 2. The Positive (PLR) and Negative Likelihood Ratio (NLR) for WINROP Algorithm eFigure 3. The Positive (PLR) and Negative Likelihood Ratio (NLR) for G-ROP Model eMethods. eTable 1. Characteristics of Included Studies of WINROP Algorithm, From High Income Countries eTable 2. Characteristics of Included Studies Evaluating WINROP Algorithm, From Low and Middle-Income Countries eTable 3. Characteristics of Included Studies Evaluating G-ROP (Postnatal Growth and ROP) eTable 4. Characteristics of Included Studies Evaluating CHOP ROP eTable 5. Characteristics of Included Studies Evaluating ROP Score Based on Cumulative Scores eTable 6. Characteristics of Included Studies Evaluating Colorado ROP [file jamanetwopen-e2135879-s001.pdf]

Supplementary Online Content

Athikarisamy S, Desai S, Patole S, Rao S, Simmer K, Lam GC. The use of postnatal weight gain algorithms to predict severe or type 1 retinopathy of prematurity: a systematic review and meta-analysis. *JAMA Netw Open.* 2021;4(11):e2135879. doi:10.1001/jamanetworkopen.2021.35879

eFigure 1. Flow Diagram for Study Selection Process

eFigure 2. The Positive (PLR) and Negative Likelihood Ratio (NLR) for WINROP Algorithm

eFigure 3. The Positive (PLR) and Negative Likelihood Ratio (NLR) for G-ROP Model

eMethods.

eTable 1. Characteristics of Included Studies of WINROP Algorithm, From High Income Countries

eTable 2. Characteristics of Included Studies Evaluating WINROP Algorithm, From Low and Middle-Income Countries

eTable 3. Characteristics of Included Studies Evaluating G-ROP (Postnatal Growth and ROP)

eTable 4. Characteristics of Included Studies Evaluating CHOP ROP

eTable 5. Characteristics of Included Studies Evaluating ROP Score Based on Cumulative Scores

eTable 6. Characteristics of Included Studies Evaluating Colorado ROP

This supplementary material has been provided by the authors to give readers additional information about their work.

© 2021 Athikarisamy S et al. *JAMA Network Open.*

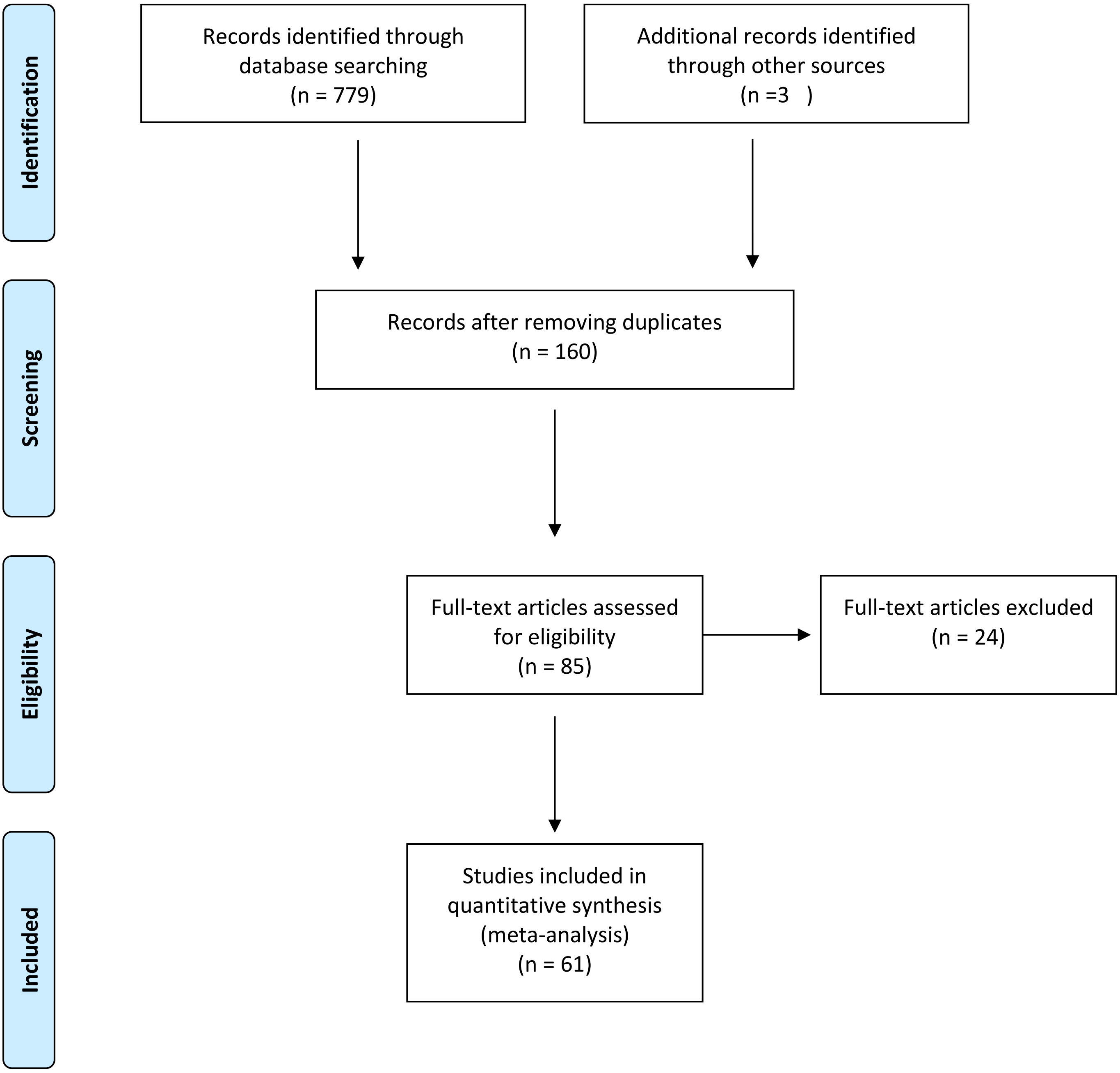

eFigure 1. Flow Diagram for Study Selection Process

© 2021 Athikarisamy S et al. *JAMA Network Open.*

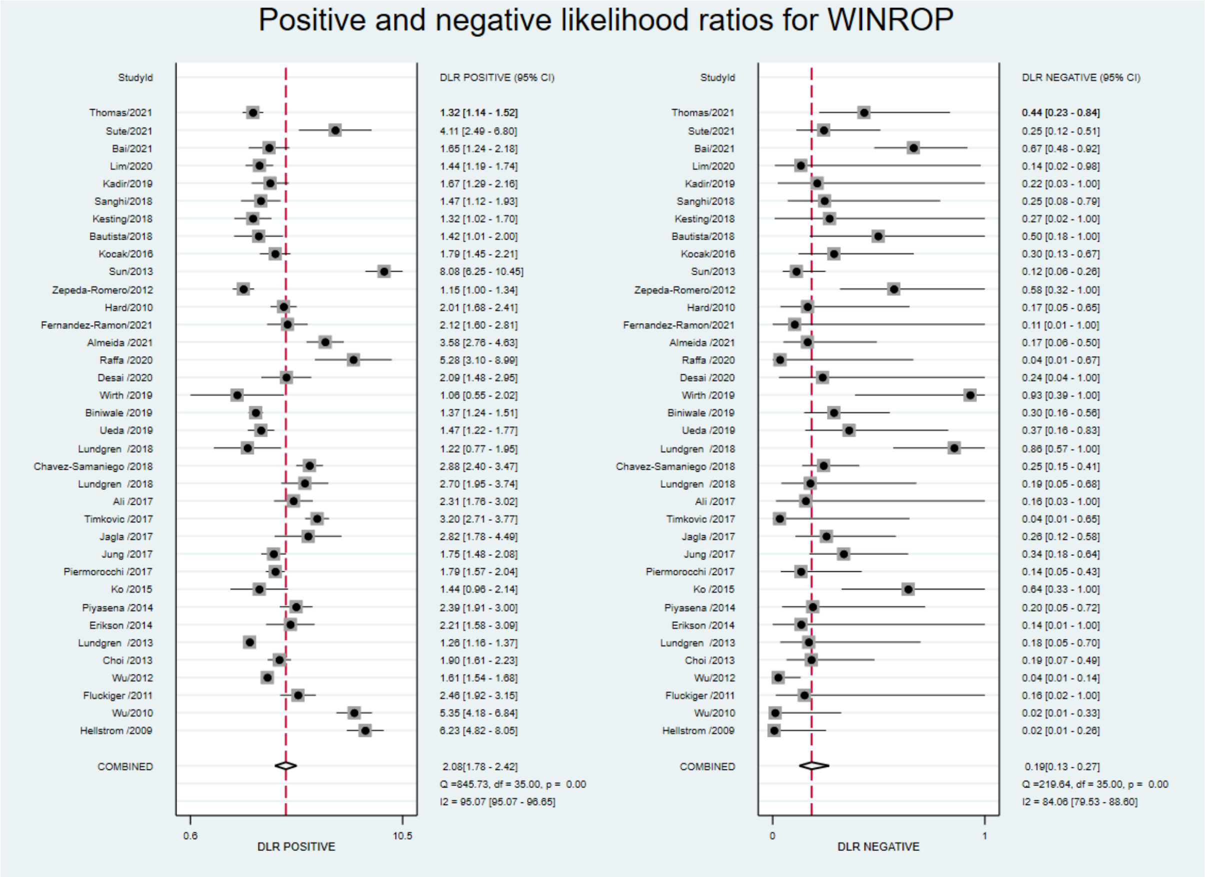

eFigure 2. The Positive (PLR) and Negative Likelihood Ratio (NLR) for WINROP Algorithm

© 2021 Athikarisamy S et al. *JAMA Network Open.*

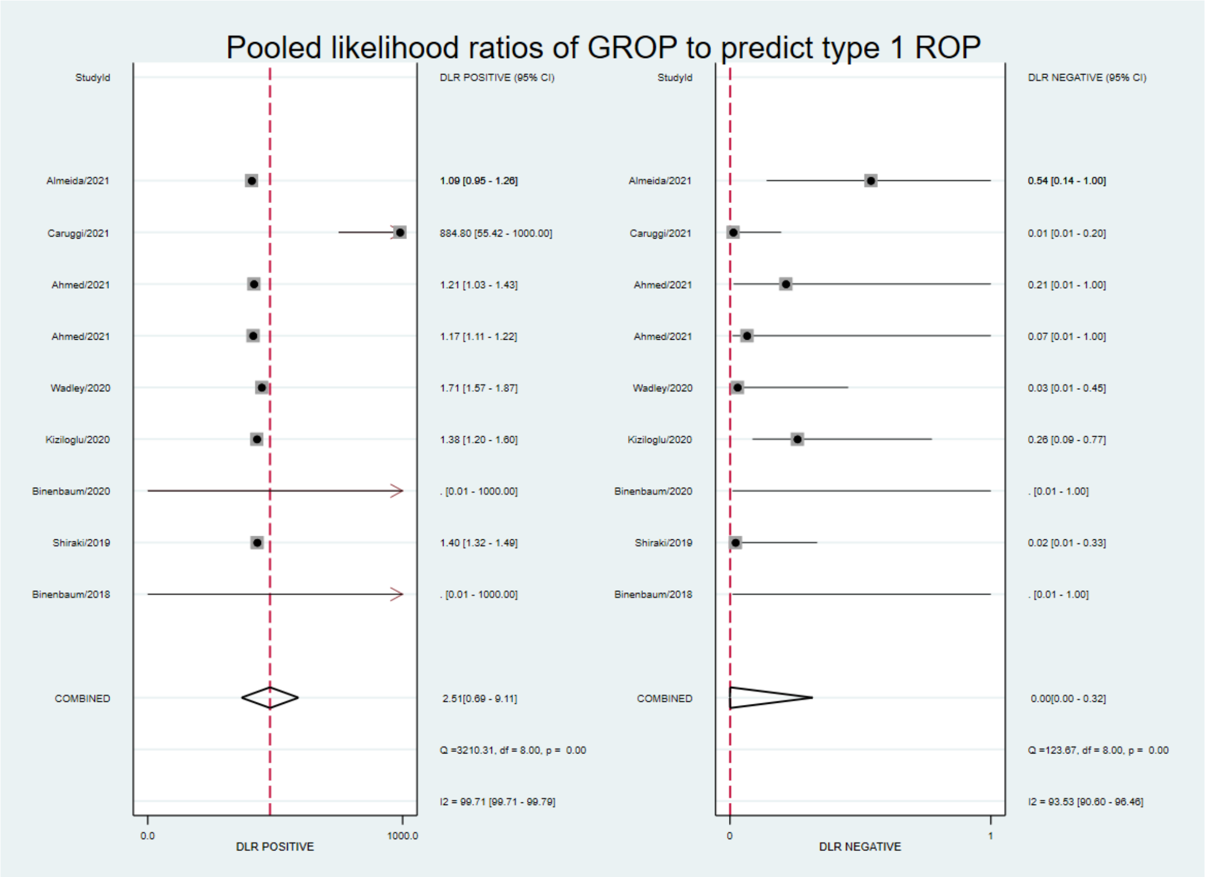

eFigure 3. The Positive (PLR) and Negative Likelihood Ratio (NLR) for G-ROP Model

© 2021 Athikarisamy S et al. *JAMA Network Open.*

eMethods.

a. Search terminology for PubMed

Mesh terminology used: (("retinopathy of prematurity"[MeSH Terms] OR ("retinopathy"[All Fields] AND "prematurity"[All Fields]) OR "retinopathy of prematurity"[All Fields]) OR "Retinopathy of Prematurity"[MeSH]) AND ((((("algorithms"[MeSH Terms] OR "algorithms"[All Fields] OR "algorithm"[All Fields]) OR ("algorithms"[MeSH Terms] OR "algorithms"[All Fields])) OR WINROP[All Fields]) OR CHOPROP[All fields]) OR ROPSCORE[All fields]) OR PINT ROP[All fields]) OR G ROP[All fields]) OR Prediction[All Fields])

b. Ovid MEDLINE(R) <1946 to September 24, 2021>

**#**	**Query**	**Results from 25 Sep 2021**
1	"diseases (non mesh)"/ or "retinopathy of prematurity"/	6,372
2	"Retinopathy of Prematurity"/	6,372
3	Weight Gain/	32,989
4	Algorithms/	274,006
5	Models, Theoretical/	157,377
6	WINROP.mp.	38
7	G-ROP.mp.	23
8	CHOP ROP.mp.	5
9	CO-ROP.mp.	6
10	ROP Score.mp.	1
11	ROPSCORE.mp.	6
12	3 or 4 or 5 or 6 or 7 or 8 or 9 or 10 or 11	447,360
13	2 and 12	148

© 2021 Athikarisamy S et al. *JAMA Network Open.*

c. Embase <1980 to 2021 September 24>

#	Query	Results from 25 Sep 2021
1	retinopathy of prematurity.mp. or retrolental fibroplasia/	12,124
2	weight gain.mp. or body weight gain/	142,837
3	algorithms.mp. or algorithm/	367,701
4	model/	462,507
5	winrop.mp.	64
6	G-ROP.mp.	72
7	CHOP ROP.mp.	14
8	CO-ROP.mp.	12
9	ROPScore.mp.	9
10	ROP Score.mp.	10
11	weight gain algorithm.mp.	2
12	2 and 3 and 4 and 5 and 6 and 7 and 8 and 9 and 10 and 11	0
13	2 or 3 or 4 or 5 or 6 or 7 or 8 or 9 or 10 or 11 or 12	959,004
14	1 and 13	628

d.

Sensitivity, specificity, positive predictive value and negative predictive value were calculated from TP (true positive), FP (false positive), FN (false negative) and TN (true negative).

In studies where TP, FP, FN, and TN were not available they were calculated by using the following formula.

1. TP = sensitivity*number with disease

2. FP= (number without disease) minus **(**specificity*number without disease)

3. FN= number with disease minus (sensitivity*number with disease)

4. TN= specificity*number without disease

© 2021 Athikarisamy S et al. *JAMA Network Open.*

eTable 1. Characteristics of Included Studies of WINROP Algorithm, From High Income Countries

Study No	Author	Year	Country	Target condition	Patient population	Study type	Total Number	True Positive	False Positive	False Negative	True Negative	Sensitivity (%)	Specificity (%)	Positive predictive value (%)	Negative predictive value (%)
1.	Hellstrom	2009	Sweden	Proliferative ROP	<32 weeks	Retrospective	353	35	50	0	268	100 (90-100)	84.28 (79.80-88.10)	41.18(35.18-47.45)	100
2.	Wu	2010	USA	Severe ROP	<32 weeks	Retrospective	318	28	53	0	237	100 (87.66-100)	81.72 (76.79-86.00)	34.57(29.29-40.26)	100
3.	Fluckiger	2011	Switzerland	Severe ROP	<32 weeks	Retrospective	376	9	134	1	232	90 (55.50-99.75)	63.39 (58.22-68.33)	6.29 (4.99-7.92)	99.57 (97.30-99.93)
4.	Wu	2012	USA and Canada	Type 1 ROP	<32 weeks	Retrospective	1706	144	957	2	603	98.63 (95.14-99.83)	38.65 (36.23-41.12)	13.08 (12.59-13.58)	99.67 (98.70-99.92)
5.	Choi	2013	South Korea	Type 1 ROP	<32 weeks	Retrospective	314	36	130	4	144	90 (76.34-97.21)	52.55 (46.46-58.59)	21.69 (19.06-24.56)	97.30 (93.38-98.92)
6.	Lundgren	2013	Sweden	Type 1 ROP	<32 weeks	Retrospective	407	45	274	2	86	95.74 (85.46-99.48)	23.89 (19.58-28.64)	14.11 (13.12-15.15)	97.73 (91.63-99.41)
7.	Erikson	2014	Sweden	Severe ROP	<32 weeks	Prospective and Retrospective	104	5	41	0	58	100 (47.82-100)	58.59 (48.24-68.40)	10.87 (8.80-13.36)	100
8.	Piyasena	2014	Scotland	Severe ROP	<32 weeks	Retrospective	410	14	144	2	250	87.50 (61.65-98.45)	63.45 (58.48-68.22)	8.86 (7.20-10.87)	99.21 (97.15-99.78)
9.	Ko	2015	Taiwan	Treatment demanding ROP	<32 weeks	Retrospective	148	11	59	6	72	64.71 (38.33-85.79)	54.96 (46.03-63.66)	15.71 (11.12-21.74)	92.31 (86.09-95.88)
10.	Piermorocchi	2017	Italy	Type 1 ROP	<30 weeks	Retrospective	377	41	173	3	160	93.18 (81.34-98.57)	48.05 (42.57-53.56)	19.16 (17.22-21.26)	98.16 (94.68-99.38)
11.	Jung	2017	North America	Severe ROP	<32 weeks	Retrospective	483	36	205	8	234	81.82 (67.29-91.81)	53.30 (48.51-58.05)	14.94 (12.89-17.25)	96.69 (93.95-98.22)
12.	Jagla	2017	Poland	Type 1 ROP	<32 weeks	Retrospective	79	22	15	5	37	81.48 (61.92-93.70)	71.15 (56.92-82.87)	59.46 (48-69.98)	88.10 (76.70-94.33)
13.	Timkovic	2017	Czechoslovakia	Type 1 ROP	<32 weeks	Retrospective	445	16	130	0	299	100 (79.41-100)	69.70 (65.11-74.01)	10.96 (9.63-12.44)	100
14.	Ali	2017	Canada	Type 1 ROP	<32 weeks	Retrospective	215	9	80	1	125	90 (55.50-99.75)	60.98 (53.93-67.69)	10.11 (7.92-12.82)	99.21 (95.10-99.88)
15.	Lundgren	2018	Sweden	Treatment demanding ROP	<31 weeks	Retrospective	127	14	36	2	75	87.50 (61.65-98.45)	67.57 (58.03-76.15)	28 (21.91-35.02)	97.40 (91.06-99.28)
16.	Chavez-Samaniego	2018	Spain	Severe ROP	<32 weeks	Retrospective	502	56	124	12	310	82.35 (71.20-90.53)	71.43 (66.93-75.64)	31.11 (27.29-35.21)	96.27 (93.90-97.74)
17.	Lundgren	2018	Sweden	Treatment demanding ROP	<31 weeks	Retrospective	142	12	46	13	71	48 (27.80-68.69)	60.68 (51.23-69.59)	20.69 (14.07-29.36)	84.52 (78.48-89.11)
18.	Ueda	2019	Japan	Type 1/Type 2 ROP	<32 weeks	Retrospective	278	27	141	5	105	84.38 (67.21-94.72)	42.68 (36.42-49.12)	16.07 (13.74-18.71)	95.45 (90.26-97.94)

© 2021 Athikarisamy S et al. *JAMA Network Open.*

19.	Biniwale	2019	USA	Type 1 ROP	<32 weeks	Retrospective	492	80	265	9	138	89.89 (81.67-95.27)	34.24 (29.62-39.10)	23.19 (21.47-25)	93.88 (89.05-96.66)
20.	Wirth	2019	France	Threshold ROP	<32 weeks	Retrospective	570	4	304	3	259	57.14 (18.41-90.10)	46 (41.83-50.22)	1.30 (0.68-2.45)	98.85 (97.34-99.51)
21.	Desai	2020	Australia	Type 1 ROP	<32 weeks	Retrospective	202	6	80	1	115	85.71 (42.13-99.64)	58.97 (51.72-65.95)	6.98 (5.04-9.59)	99.14 (94.91-99.86)
22.	Raffa	2020	Saudi Arabia	Type 1 ROP	<32 weeks	Retrospective	75	13	11	0	51	100 (75.29-100)	82.26 (70.47-90.80)	54.17 (40.88-66.89)	100
23.	Almeida	2021	Portugal	Type 1 ROP	< 32 weeks	Retrospective	311	20	70	3	218	86.96 (66.41-97.22)	75.69 (70.32-80.53)	22.22 (18.08-27)	98.64 (96.19-99.52)
24.	Fernández-Ramón	2021	Spain	Severe ROP	<32 weeks	Retrospective	109	7	45	0	57	100 (59.04-100)	55.88 (45.71-65.71)	13.46 (11.11-16.21)	100

© 2021 Athikarisamy S et al. *JAMA Network Open.*

eTable 2. Characteristics of Included Studies Evaluating WINROP Algorithm, From Low and Middle-Income Countries

Study No	Author	Year	Country	Target condition	Patient population	Study type	Total Number	True Positive	False Positive	False Negative	True Negative	Sensitivity (%)	Specificity (%)	Positive predictive value (%)	Negative predictive value (%)
1.	Hard	2010	Brazil	Severe ROP	<32 weeks	Retrospective	366	19	155	2	190	90.48 (69.62-98.83)	55.07 (49.65-60.40)	10.92 (9.28-12.81)	98.96 (96.20-99.72)
2.	Zepeda-Romero	2012	Mexico	Type 1 ROP	<32 weeks	Retrospective	192	83	69	15	25	84.69 (76.01-91.17)	26.60 (18.01-36.71)	54.61 (50.92-58.24)	62.50 (48.42-74.74)
3.	Sun	2013	China	Severe ROP	<32 weeks	Retrospective	590	50	59	6	475	89.29 (78.12-95.97)	88.95 (85.98-91.48)	45.87 (39.59-52.29)	98.75 (97.38-99.41)
4.	Kocak	2016	Turkey	Type 1 ROP	<32 weeks	Retrospective	223	27	90	5	101	84.38 (67.21-94.72)	52.88 (45.54-60.13)	23.08 (19.53-27.05)	95.28 (89.93-97.86)
5.	Bautista	2018	Philippines	Treatment demanding ROP	---	----	137	10	67	3	57	76.92 (46.19-94.96)	45.97 (36.99-55.15)	12.99 (9.61-17.32)	95 (87.37-98.12)
6.	Kesting	2018	South Africa	Severe ROP	<32 weeks	Retrospective	220	5	150	0	65	100 (47.82-100)	30.23 (24.17-36.85)	3.23 (2.96-3.51)	100
7.	Sanghi	2018	India	Type 1 ROP	<32 weeks	Retrospective	70	28	24	3	15	90.32 (74.25-97.96)	38.46 (23.36-55.38)	53.85 (47.02-60.53)	83.33 (61.37-94.02)
8.	Kadir	2019	Malaysia	Type 1 ROP	<32 weeks	Prospective	151	9	76	1	65	90 (55.50-99.75)	46.10 (37.68-54.69)	10.59 (8.39-13.28)	98.48 (90.94-99.76)
9.	Lim	2020	Malaysia	Type 1 ROP	<32 weeks	Retrospective	92	20	47	1	24	95.24 (76.18-99.88)	33.80 (23-46.01)	29.85 (26-34.01)	96 (77.52-99.40)
10.	Bai	2021	China	Type 1 ROP	<32 weeks	Retrospective	432	28	130	22	252	56 (41.25-70.01)	65.97 (60.98-70.71)	17.72 (13.97-22.22)	91.97 (89.26-94.04)
11.	Sute	2021	India	Type 1 ROP	<32 weeks	Prospective	102	24	14	6	58	80 (61.43-92.29)	80.56 (69.53-88.94)	63.16 (50.90-73.92)	90.62 (82.41-95.23)
12.	Thomas	2021	India	Type 1 ROP	<32 weeks	Ambispective	382	43	212	8	119	84.31 (71.41-92.98)	35.95 (30.78-41.38)	16.86 (14.95-18.97)	93.70 (88.57-96.62)

© 2021 Athikarisamy S et al. *JAMA Network Open.*

eTable 3. Characteristics of Included Studies Evaluating G-ROP (Postnatal Growth and ROP)

Study No	Author	Year	Country	Study type	Target Disease	Total Number	Target disease number	True Positive	False Positive	False Negative	True Negative	Sensitivity (%)	Specificity (%)	Positive predictive value (%)	Negative predictive value (%)
1.	Binenbaum	2018	USA	Retrospective	Type 1 ROP	7483	459	459	NA	0	NA	100.00 (99.20-100.00)	NA	NA	NA
2.	Shiraki	2019	Japan	Retrospective	Treatment requiring ROP	537	81	81	324	0	132	100 .00 (95.4-100)	28.9 (24.9-33.2)	20.00 (19.08-20.95)	100
3.	Binenbaum	2020	USA	Prospective	Type 1 ROP	3981	219	219	NA	0	NA	100.00 (98.33 -100.00)	NA	NA	NA
4.	Yabas Kiziloglu	2020	Turkey	Retrospective	Treatment requiring ROP	242	34	31	137	3	71	91.2 (76.32-98.14)	34.13 (27.72-41.01)	18.45 (16.39-20.71)	95.95 (88.77-98.61)
5.	Wadley	2020	USA	Retrospective	Type 1 ROP	484	40	40	256	0	188	100.00 (91.19-100.00)	42.34 (37.70-47.09)	13.51 (12.61 -14.47)	100
6.	Ahmed	2021	Egypt and UK	Retrospective	Type 1 ROP	605	60	60	458	0	87	100 .00 (94.04-100.00)	15.96 (12.99-19.31)	11.58 (11.21-11.96)	100
7.	Caruggi	2021	Italy	Retrospective	Type 1 ROP	475	39	39	0	0	447	100 (90.97- 100.00)	100 (99.18- 100.00)	100	100
8.	Almeida	2021	Portugal	Retrospective	Type 1 ROP	313	22	20	242	2	49	90.9 (70.8-99.0)	16.7 (8.9-27.3)	25.0 (22.0-28.3)	85.7 (59.2-96.12)

NA: (Data) Not available

For studies 1 & 3 only sensitivity values could be calculated from the given data.

© 2021 Athikarisamy S et al. *JAMA Network Open.*

eTable 4. Characteristics of Included Studies Evaluating CHOP ROP

Study No	Author	Year	Country	Study type	Cut off point	Target Disease	Total Number	True Positive	False Positive	False Negative	True Negative	Sensitivity (%)	Specificity (%)	Positive predictive value (%)	Negative predictive value (%)
1.	Binenbaum	2012	USA	Retrospective	0.0140	Type 1 ROP	524	20	237	0	267	100 (83-100)	53 (49-57.4)	7.78 (7.14 – 8.47)	100 (98-100)
2.	Binenbaum	2017	USA	Retrospective	0.0140	Type 1 ROP	459	450	4495	9	2529	98.5 (96.9-99.3)	36.4 (34.88-37.14)	9.10 (8.92-9.28)	99.65 (99.32-99.81)
					0.0026	Type 1 ROP	459	459	6462	0	562	100 (99.2-100)	7.8 (7.38-8.66)	6.63 (6.59-6.67)	100
					0.0034	Type 1 ROP	459	459	6181	0	843	100 (99.2-100)	12 (11.25-12.78)	6.91 (6.86-6.97)	100
3.	Piermarocchi	2017	Italy	Retrospective	0.010	Type 1 ROP	399	44	142	0	213	100 (92-100)	60.3 (55.1-65.2)	23.8 (18.2-30.4)	100 (98.2-100)
					0.016	Type 1 ROP	399	44	114	0	241	100 (92-100)	67.6 (62.6-72.3)	27.7 (21.3-35.1)	100 (98.4-100)
4.	Doshi	2019	India	Retrospective	0.0140	Type 1 ROP	191	8	45	4	134	66.67 (34.89-90.08)	74.58 (67.50-80.81)	15.09 (9.97-22.20)	97.06 (93.65-98.66)
					0.010	Type 1 ROP	191	12	88	0	91	100 (73.54-100)	51.40 (43.83-58.92)	12.12 (10.61-13.82)	100
5.	Sun	2021	China	Retrospective	0.014	Type 1 ROP	180	180	2692	0	715	100 (97.97-100)	21.4 (19.63-22.39)	6.27 (6.17-6.37)	100
					0.0026	Type 1 ROP	180	180	3339	0	68	100 (97.97-100)	2.7 (1.55-2.52)	5.12 (5.09-5.14)	100
					0.0034	Type 1 ROP	180	180	3271	0	136	100 (97.97-100)	4.3 (3.36-4.70)	5.22 (5.18-5.25)	100
6.	Thomas	2021	India	Ambispective	0.014	Type 1 ROP	382	28	96	23	235	54.90 (40.34-68.87)	71.00 (65.78-75.83)	22.58 (17.76- 28.26)	91.09 (88.22- 93.31)

© 2021 Athikarisamy S et al. *JAMA Network Open.*

eTable 5. Characteristics of Included Studies Evaluating ROP Score Based on Cumulative Scores

Study No	Author	Year	Cut off point for the cumulative score	Country	Study type	Target Disease	Total Number	True Positive	False Positive	False Negative	True Negative	Sensitivity (%)	Specificity (%)	Positive predictive value (%)	Negative predictive value (%)
1.	Eckert	2012	14.5	Brazil	Prospective	Severe ROP	474	23	198	1	25	95.83 (78.88-99.89)	56 (51.28-60.64)	10.41 (9.23-11.72)	99.6 (97.36-99.94)
2.	Piermarocchi	2017	14.5	Italy	Retrospective	Type 1 ROP	399	44	174	0	181	100 (92.0-100)	51.3 (46.1-56.4)	20.3 (15.5-26.1)	100 (97.9-100)
3.	Piermarocchi	2017	15.8	Italy	Retrospective	Type 1 ROP	399	44	110	0	245	100 (92.0-100)	69.0 (64.0-73.6)	28.5 (22.0-36.2)	100 (94.5-100)
4.	Lucio	2018	16.6	Brazil	Prospective	Severe ROP	181	21	27	1	132	95.4 (86.7-100)	83.6 (77.9-89.4)	44.7 (30.5-58.9)	99.2 (97.8-100)
5.	Figueiredo	2020	14.7	Portugal	Retrospective	Severe ROP	239	12	139	0	88	100 (71.3-100.0)	39.3 (29.8-49.7)	18.2 (10.1-30.0)	100.0 (87.7-100.0)
6.	Gulkas	2021	14.5	Turkey	Retrospective	Type 1 ROP	131	19	19	0	93	100 (82.35-100)	83.04 (74.78-89.47)	50 (39.90-60.10)	100
7.	Sun	2021	13.3	China	Retrospective	Severe ROP	3587	90	443	90	2964	50 (42.47- 57.53)	87 (85.82- 88.11)	16.89 (14.63- 19.41)	97.05(96.60-97.44)
8	Thomas	2021	14.5	India	Retrospective	Type 1 ROP	382	37	109	14	222	72.5 (58.26-84.11)	67.07 (61.72-72.11)	25.34 (21.27-29.90)	94.07 (90.98-96.14)

© 2021 Athikarisamy S et al. *JAMA Network Open.*

eTable 6. Characteristics of Included Studies Evaluating Colorado ROP

Study No	Author	Year	Net weight gain between birth and 1 month of age	Country	Study type	Target Disease	Total Number	True Positive	False Positive	False Negative	True Negative	Sensitivity (%)	Specificity (%)	Positive predictive value (%)	Negative predictive value (%)
1.	Cao CO-ROP model	2016	< 650g	USA	Retrospective	Type 1 ROP	499	30	351	0	118	100 (92.1-100)	33.7 (28.7-39.1)	7.87 (7.50-8.26)	100
2.	Cao (high grade CO-ROP model)	2016	< 400g	USA	Retrospective	Type 1 ROP	499	30	240	0	229	100 (92.1-100)	59.6 (54.1-65.0)	11.11 (10.27-12.01)	100
3.	Cao CO-ROP model	2016	< 650g	USA	Retrospective	Type 1 ROP	858	82	571	1	204	98.8 (93.5-100)	26.32 (23.25-29.57)	12.56 (12.04-13.10)	99.51 (96.66-99.93)
4.	Huang	2017	< 650g	USA	Retrospective	Type 1 ROP	374	27	241	2	104	93.1 (77.23-99.15)	30.14 (25.35-35.29)	10.07 (9.03-11.22)	98.11 (93.11-99.50)
5.	McCourt	2018	< 650g	USA & Canada	Retrospective	Type 1 ROP	6351	338	2267	14	3732	96.02 (93.4-97.6)	62.21 (60.97-63.44)	12.98 (12.54-13.42)	99.63 (99.38-99.78)

© 2021 Athikarisamy S et al. *JAMA Network Open.*
